# Loss of function of *CMPK2* causes mitochondria deficiency and brain calcification

**DOI:** 10.1038/s41421-022-00475-2

**Published:** 2022-11-29

**Authors:** Miao Zhao, Hui-Zhen Su, Yi-Heng Zeng, Yuan Sun, Xin-Xin Guo, Yun-Lu Li, Chong Wang, Zhi-Yuan Zhao, Xue-Jing Huang, Kai-Jun Lin, Zi-Ling Ye, Bi-Wei Lin, Shunyan Hong, Jitan Zheng, Yao-Bin Liu, Xiang-Ping Yao, Dehao Yang, Ying-Qian Lu, Hai-Zhu Chen, Erwei Zuo, Guang Yang, Hong-Tao Wang, Chen-Wei Huang, Xiao-Hong Lin, Zhidong Cen, Lu-Lu Lai, Yan-Ke Zhang, Xi Li, Tianmin Lai, Jingjing Lin, Dan-Dan Zuo, Min-Ting Lin, Chia-Wei Liou, Qing-Xia Kong, Chuan-Zhu Yan, Zhi-Qi Xiong, Ning Wang, Wei Luo, Cui-Ping Zhao, Xuewen Cheng, Wan-Jin Chen

**Affiliations:** 1 Department of Neurology, the First Affiliated Hospital, Institute of Neuroscience, Fujian Key Laboratory of Molecular Neurology, Fujian Medical University, Fuzhou, Fujian China; 2 Neurology, Qilu Hospital, Cheeloo College of Medicine, Shandong University, Qingdao, Shandong China; 3 Qingdao Key Lab of Mitochondrial Medicine, Qilu Hospital, Cheeloo College of Medicine, Shandong University, Qingdao, Shandong China; 4 Department of Neurology, Sanming Hospital of Integrated Traditional and Western Medicine, Sanming, Fujian China; 5 Department of Neurology, The Second Affiliated Hospital, Zhejiang University School of Medicine, Hangzhou, Zhejiang China; 6 Center for Animal Genomics, Agricultural Genome Institute at Shenzhen, Chinese Academy of Agricultural Sciences, Shenzhen, Guangdong China; 7 Center for Excellence in Brain Science and Intelligence Technology, Institute of Neuroscience and State Key Laboratory of Neuroscience, Chinese Academy of Sciences, Shanghai, China; 8 University of Chinese Academy of Science, Beijing, China; 9 Department of Neurology, Affiliated Hospital of Jining Medical University, Jining, Shandong China; 10 Department of Neurology, Affiliated Hospital of Xuzhou Medical University, Xuzhou, Jiangsu China; 11 Department of Neurology, Kaohsiung Chang Gung Memorial Hospital and Chang Gung University College of Medicine, Kaohsiung, Taiwan, China; 12 Lingang Laboratory, Shanghai Center for Brain Science and Brain-Inspired Intelligence Technology, Shanghai, China

**Keywords:** DNA metabolism, Mechanisms of disease

## Abstract

Brain calcification is a critical aging-associated pathology and can cause multifaceted neurological symptoms. Cerebral phosphate homeostasis dysregulation, blood-brain barrier defects, and immune dysregulation have been implicated as major pathological processes in familial brain calcification (FBC). Here, we analyzed two brain calcification families and identified calcification co-segregated biallelic variants in the *CMPK2* gene that disrupt mitochondrial functions. Transcriptome analysis of peripheral blood mononuclear cells (PBMCs) isolated from these patients showed impaired mitochondria-associated metabolism pathways. In situ hybridization and single-cell RNA sequencing revealed robust *Cmpk2* expression in neurons and vascular endothelial cells (vECs), two cell types with high energy expenditure in the brain. The neurons in *Cmpk2-*knockout (KO) mice have fewer mitochondrial DNA copies, down-regulated mitochondrial proteins, reduced ATP production, and elevated intracellular inorganic phosphate (Pi) level, recapitulating the mitochondrial dysfunction observed in the PBMCs isolated from the FBC patients. Morphologically, the cristae architecture of the *Cmpk2-*KO murine neurons was also impaired. Notably, calcification developed in a progressive manner in the homozygous *Cmpk2*-KO mice thalamus region as well as in the *Cmpk2-*knock-in mice bearing the patient mutation, thus phenocopying the calcification pathology observed in the patients. Together, our study identifies biallelic variants of *CMPK2* as novel genetic factors for FBC; and demonstrates how CMPK2 deficiency alters mitochondrial structures and functions, thereby highlighting the mitochondria dysregulation as a critical pathogenic mechanism underlying brain calcification.

## Introduction

Brain calcification is increasingly recognized as a representative pathological hallmark linked to neurodegeneration and aging, with an age-correlated prevalence from about 1% in young people to over 20% among the elderly^1,2^. Genetic mutations have been reported as triggering or risk factors for a large proportion of bilateral brain calcification cases^3^. Currently, no effective therapy is clinically available for the brain calcification treatment. The development of effective therapies had been largely hindered by the incomplete mapping of their genetic basis and the underlying mechanism. For example, as a classical type of inheritable familial brain calcification (FBC), primary familial brain calcification (PFBC), is a monogenic neurodegenerative disorder characterized by bilateral calcification that occurs predominantly in the basal ganglia, thalamus, and cerebellum, and occasionally in subcortical white matter^1,4–6^. Although two decades of human genetic studies have revealed mutations in *SLC20A2*, *XPR1*, *PDGFB*, *PDGFRB, MYORG*, and *JAM2*^7–13^ as causative factors for PFBC, these six genes can only explain half of PFBC families^4,14^. Therefore, the identification of new genetic factors for the remaining FBC is still in urgent need to combat brain calcification.

Mitochondrial function impairment is tightly associated with neurodegenerative diseases like Alzheimer’s disease and Parkinson’s disease^15^. Given the causal link between mitochondria stress and neurodegeneration is largely established, the significance of the upstream molecular programs, especially those destructing normal mitochondria DNA synthesis, should be further addressed. Moreover, brain calcifications were also observed among a series of mitochondriopathies (e.g., Kearn-Sayre syndrome, Leigh syndrome)^16–18^. Dysregulation of mitochondria DNA synthesis is a critical trigger for the activation of interferon response pathway^19,20^, which could sufficiently induce brain calcification in both animal model and in patients of Aicardi–Goutières syndrome^19^. Therefore, the convergence of these pathogenesis suggests mitochondria DNA homeostasis may be a crucial hub in maintaining brain health. However, the underlying mechanism between mitochondria dysfunction and brain calcification is not yet unraveled.

Here, we identified biallelic variants in the gene *UMP-CMP kinase 2* (*CMPK2*, NM_207315) from two brain calcification families based on whole-exome sequencing (WES). Consistent with the relative high expression levels of *Cmpk2* mRNA in neurons and in vascular endothelial cells (vECs), abnormal mitochondrial functions were parallelly found in transcriptome analysis of isolated patients’ peripheral blood mononuclear cells (PBMCs), as well as the analyses of neurons from *Cmpk2-*knockout (KO) mouse brains. In both *Cmpk2*-KO mice and patient-mutation knock-in (KI) mice subjected to histochemical and micro-CT analyses of brain sections, progressive calcification deposits were observed in the thalamus, phenocopying the calcification pathology observed in the patients. Collectively, our study demonstrates how the loss of *Cmpk2* impairs mitochondrial function and leads to brain calcifications in humans and animal models.

## Results

### Clinical manifestations of patients with brain calcification

**Fig. 1:**
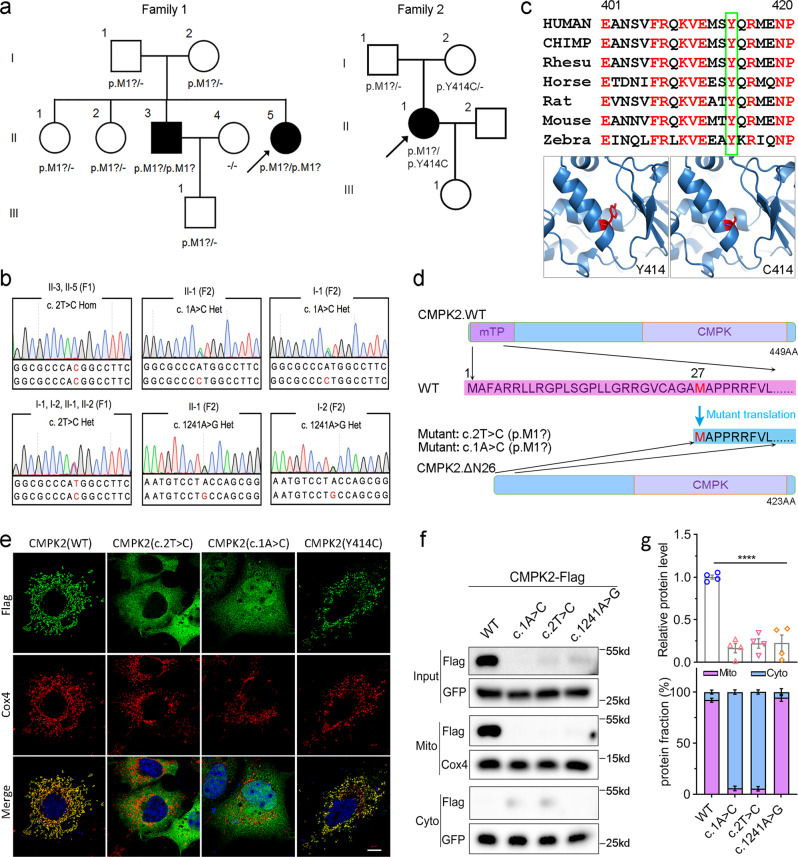
*CMPK2* variants in FBC patients. **a** The pedigrees of the brain calcification families (Family 1 and Family 2) with *CMPK2* variants. Filled or empty symbols represent individuals with or without brain calcification. “+/−” indicates heterozygous variant carriers, “−/−” indicates individuals without this variant. The arrows represent the probands. **b** Sequencing chromatograms showing *CMPK2* c.2 T > C in Family 1, and *CMPK2* c.1 A > C and c.1241 A > G in Family 2, with the reference allele below. The variants are marked in red. het heterozygous, hom homozygous. **c** Homologous alignment of the CMPK2 protein sequence bearing the core domain α7b helix that includes p.Y414, and the protein conformation change of Y414C. **d** A schematic diagram of the initiation codon loss of *CMPK2*. *CMPK2* (c.1 A > C p.M1?; c.2 T > C, p.M1?) mRNA transcription could perhaps be re-initiated from a subsequent ATG codon (p.M27), leading to loss of the mTP in the CMPK2 protein (△N26). mTP mitochondrial targeting peptide sequence, CMPK CMP-UMP kinase domain. **e** Immunofluorescence analysis of subcellular localization of the CMPK2 protein. Cos-7 cells were transfected with Flag-tagged CMPK2 wild-type (WT), c.1 A > C, c.2 T > C, and c.1241 A > G mutant plasmids, and immune-stained with antibodies against Flag (green) and the mitochondria marker COX IV (red). DAPI (blue) was used for nuclear staining. Scale bar, 5 μm. **f** Western blot analysis for the mitochondrial and cytoplastic expression of CMPK2. Primary cultured rat neurons were transfected with Flag-tagged CMPK2-WT, c.1 A > C, c.2 T > C, and c.1241 A > G plasmids. The CMPK2 protein quantitative statistics by Image J were displayed in **g**, ****P < 0.0001.

To explore underlying genetic causes for the genetically undetermined 45 FBC families, we initially focused on a family with autosomal recessive inheritance. The two affected siblings (II-3 and II-5) in Family 1 exhibited obvious calcification in the bilateral globus pallidus, thalamus, and cerebellum (Fig. 1a; Supplementary Fig. S1). For II-5, bulk calcification was also apparent in the caudate nucleus, periventricular white matter, and the cerebral cortex, with a total calcification (TCS) value of 52. At the first visit (40-year-old), II-5 exhibited moderate motor dysfunction, speech impairment, and cognitive deficit, as well as forced laughter and crying. Two years later, she suffered progressively exacerbated symptoms, with impairment in language-mediated communication, difficulty in standing, and disability in self-care. At that time, she was unable to participate in the standard scoring measurement, indicating substantial further cognitive decline. Her ECG result showed mild pulmonary arterial hypertension, and cerebrospinal fluid (CSF) examination revealed a slightly increased CSF white blood cell (WBC) count (value 10), a low level of CSF lactate (2.2 mmol/L), and a normal level of CSF Pi. Echocardiography, abdominal ultrasonography, anti-nuclear antibodies, blood, and urine examinations were all within normal levels (Table 1).

**Table 1:** A summary of genetic and clinical features in patients with *CMPK2* mutations.

	Family 1			Family 2	
	II-3		II-5	II-1	
Genetic information					
Gene	*CMPK2*			*CMPK2*	
Transcript	NM_207315			NM_207315	
Variant Coordinate^a^	Chr2:6865695			Chr2:6865696	Chr2:6849959
Nucleotide Change	c.2 T > C			c.1 A > C	c.1241 A > G
Amino Acid Alteration	p.M1?			p.M1?	p.Y414C
Mutation Type	Start lost, homozygous			Start lost, heterozygous	Missense, heterozygous
gnomAD genomes (all)	Absent			Absent	Absent
gnomAD exomes (all)	Absent			Absent	4.06336e-06 (1/246102)
ExAC	Absent			Absent	Absent
1000 Genomes	Absent			Absent	Absent
Mutation Taster	Disease causing			Disease causing	Disease causing
SIFT	NA			NA	Deleterious
PolyPhen-2	NA			NA	Probably damaging
M-CAP	Possibly pathogenic			Possibly pathogenic	Damaging
CADD_PHRED	21.8			23.3	29.7
ACMG classification^*^	Pathogenic (PS3 + PM1 + PM2 + PM3+PM4 + PP1 + PP3 + PP4)			Pathogenic (PS3 + PM1 + PM2 + PM3+PM4 + PP1 + PP3 + PP4)	Pathogenic (PS3 + PM2 + PM3 + PP1 + PP3 + PP4)
Clinical features					
Sex	Male	Female		Female	
Age at exam	44	40		39	
Cognitive Impairment	−	+		+	
Ataxia	−	+		+	
Dysarthria	−	+		+	
Seizure	−	−		−	
Headache	−	−		−	
Stroke-like episodes	−	−		−	
Serum calcium (mmol/L)	2.22	2.48		2.16	
Serum phosphorous (mmol/L)	1	1.06		1.34	
PTH (ng/L)	NA	45.81		46.55	
Antinuclear Antibodies	−	−		+	
CSF WBC count (×10^6^/L)	NA	10		NA	
CSF lactic acid (mmol/L)	NA	2.2		NA	
CSF Pi (mmol/L)	NA	0.46		NA	
ECG	Normal	Normal		NA	
EEG	NA	NA		Normal	
EMG	NA	NA		Normal	
Echocardiography	mild pulmonary hypertension	Normal		NA	
Abdominal Ultrasonography	Normal	Normal		NA	
TCS^b^	20	52		57	
MDC^c^	1	4		1	

*PTH* parathyroid hormone, *CSF* cerebrospinal fluid, *WBC* white blood cell, *ECG* Electrocardiograph, *EEG* Electroencephalogram, *EMG* Electromyography, *TCS* total calcification score, *NA* not available.

Normal laboratory test results were as follows: serum calcium 2.15–2.55 mmol/L, serum phosphorous 0.87–1.45 mmol/L, PTH 15–65 ng/L, CSF WBC count 0–8×10^6^/L, CSF lactic acid 0.7–2.1 mmol/L, CSF Pi 0.288–0.667 mmol/L.

^a^Position on Genome Reference Consortium human genome build 38 (GRCh38).

^b^TCS, Total calcification score^2^.

^c^MDC, Mitochondrial disease criteria^29^: score 1: mitochondrial disorder unlikely; score 2 to 4: possible mitochondrial disorder; score 5 to 7: probable mitochondrial disorder; score 8 to 12: definite mitochondrial disorder.

^*^ACMG-AMP guidelines are formally applied to known Mendelian genes, and we classified these variants for the convenience of further use of these data.

Note that although II-5’s brother (II-3) (44-year-old at the first visit) had a TCS value of 20, he was asymptomatic, and his neurological examination revealed no abnormalities. Both II-3 and II-5 showed normal serum calcium and phosphate levels (Table 1). There is no evidence indicating a consanguineous marriage of I-1 and I-2. None of other examined Family 1 members (I-1, I-2, II-1, II-2, and III-1) had any apparent brain calcification above the physiological threshold (Supplementary Fig. S1) or neurological symptoms.

Subsequently, we found another autosomal recessive FBC family whose proband presented similar brain calcification pattern (TCS 57) and clinical manifestations as II-5 in Family 1 (Fig. 1a, Table 1; Supplementary Fig. S1). This 39-year-old woman exhibited progressive slurred speech and impaired cognition (MMSE score 21 (bachelor degree), MoCA score 24) for 7 years; and developed aggravating movement disorders, as well as urine and fecal incontinence during the last two years. Her EEG, EMG, serum calcium, phosphate, PTH, and other biochemical indicators were normal, but with an elevation in her anti-SSA level (Table 1). Neither of her parents reported any presentations potentially linked to brain calcifications.

### Identification of the *CMPK2* biallelic variants

To seek novel genetic factor(s) potentially underlying the development of the FBC, we performed WES for I-1, I-2, II-3, and II-5 of Family 1. Assuming the hypothesis of an autosomal recessive inheritance pattern, we employed strategies to search for biallelic mutations present in the two patients but absent from the unaffected siblings. In this way, we identified two candidate genes harboring biallelic variants (*CMPK2* (c.2 T > C, p.M1?) and *LPIN1* (c.1100 C > G, p.P367R)) with complete co-segregation between mutations and the brain calcification phenotype. We also screened for the copy number variants, deep intronic variants, and short tandem repeat variants of known FBC causing genes of II-5 in Family 1 by whole-genome sequencing (WGS), but no suspicious variants were detected. The amino acid of the missense variant of *LPIN1* (c.1100 C > G, p.P367R) in our family was not conserved across different species (Supplementary Fig. S2), and the pathogenicity of this variant was predicted below the harmful threshold (Supplementary Table S1). On the contrary, the homozygous null variant of *CMPK2* (c.2 T > C, p.M1?) was predicted as a loss-of-function variant.

WES analysis of Family 2 with a recessive inheritance also revealed compound heterozygous variants in the *CMPK2* gene (c.1 A > C, p.M1?; c.1241 A > G, p.Y414C) in II-1 with severe brain calcification. Sanger sequencing was then performed, which confirmed the co-segregation of biallelic *CMPK2* variants with the brain calcification phenotype in the two families (Fig. 1b). Notably the three *CMPK2* variants were absent (c.1 A > C and c.2 T > C) or had an extremely low frequency (c.1241 A > G) in ESP, gnomAD, ExAC, and 1000 Genomes databases (Table 1), and were not detected in our 500 ethnically matched healthy controls. Also, all the three variants were evaluated as “pathogenic” according to ACMG standards and guidelines (Table 1). Additionally, a total of 43 heterozygous stop-gain, frameshift, and start-lost variants of *CMPK2* are present in gnomAD with very low allele frequencies (Table 2).

**Table 2:** Stop-gained, frameshift, and start-lost variants of *CMPK2* in gnomAD database.

Chromosome Positions^a^	rsIDs	Transcript Consequence^b^	Protein Consequence	Annotation	Total population		East Asian population	
					Allele Frequency^c^	Homozygote Count	Allele Frequency^d^	Homozygote Count
2:6851542	rs776359222	c.1126_1133del	p.Thr376SerfsTer2	frameshift	6.577E-06	0	0	0
2:6851587	rs1188711213	c.1088del	p.Pro363GlnfsTer15	frameshift	6.5699E-06	0	0.00019253	0
2:6851595	rs1177412695	c.1081 C > T	p.Gln361Ter	stop_gained	6.5715E-06	0	0	0
2:6851628	rs200083396	c.1048 C > T	p.Gln350Ter	stop_gained	6.5721E-06	0	0.00019275	0
2:6851632	–	c.1043dup	p.Leu349SerfsTer22	frameshift	6.6141E-06	0	0	0
2:6851660	–	c.1015del	p.Tyr339MetfsTer3	frameshift	6.5719E-06	0	0	0
2:6861364	rs200690985	c.812 C > A	p.Ser271Ter	stop_gained	0.00024969	0	0	0
2:6861364	rs200690985	c.812 C > G	p.Ser271Ter	stop_gained	6.5707E-06	0	0	0
2:6861368	rs757743769	c.808 C > T	p.Gln270Ter	stop_gained	1.3146E-05	0	0	0
2:6863518	–	c.736 C > T	p.Gln246Ter	stop_gained	6.5701E-06	0	0	0
2:6863524	rs773169808	c.710_729del	p.Leu237ProfsTer22	frameshift	6.5739E-06	0	0	0
2:6865075	rs1448087587	c.621del	p.Leu207PhefsTer62	frameshift	6.5697E-06	0	0	0
2:6865141	rs757223880	c.556 C > T	p.Gln186Ter	stop_gained	6.5685E-06	0	0	0
2:6865304	rs894719630	c.391_392del	p.Phe131ProfsTer8	frameshift	6.571E-06	0	0	0
2:6865317	rs774163844	c.379del	p.Ala127HisfsTer142	frameshift	6.5706E-06	0	0	0
2:6865354	–	c.326_342del	p.Arg109ProfsTer25	frameshift	6.5738E-06	0	0.00019283	0
2:6865396	–	c.301 C > T	p.Gln101Ter	stop_gained	6.5822E-06	0	0	0
2:6865397	rs1352574559	c.299_300insG	p.His100GlnfsTer40	frameshift	4.607E-05	0	0	0
2:6865412	–	c.257_284dup	p.His95GlnfsTer54	frameshift	6.5858E-06	0	0	0
2:6865439	rs1485742827	c.230_257del	p.Val77GlyfsTer183	frameshift	6.59E-06	0	0	0
2:6865476	rs1178431768	c.221 C > A	p.Ser74Ter	stop_gained	6.5977E-06	0	0	0
2:6865582	–	c.114del	p.Asp39ThrfsTer9	frameshift	6.5963E-06	0	0	0

^a^Position on Genome Reference Consortium human genome build 38 (GRCh38).

^b^The accession number for *CMPK2* is NM_207315.

^c^The frequency of the mutation in total population was calculated by mutated allele number/total allele number in parentheses.

^d^The frequency of the mutation in east Asian population was calculated by mutated allele number/total allele number in parentheses. gnomAD, Genome Aggregation Database.

The c.1 A > C (p.M1?) and c.2 T > C (p.M1?) variants cause loss of the initiation codon of the *CMPK2* open reading frame, suggesting that any translation of such a mutant *CMPK2* mRNA transcript could be disrupted, or otherwise re-initiated from a subsequent ATG codon (c.79_81, p.M27) (Fig. 1d). Such a scenario would result in truncation of CMPK2’s N-terminal domain (1–26 aa), which is known to be essential for its mitochondria-targeting^21^. The missense variant c.1241 A > G led to the mutation of the highly conserved tyrosine to cysteine (p.Y414C) within the α7b helix of the CORE domain in the CMPK2 protein (Fig. 1c; Supplementary Fig. S3). This tyrosine residue likely provides critical structural support to nearby R407 and R416, which functions as positively-charged docking sites for the negatively charged phosphate in ATP^22^, and its mutation to cysteine may disrupt the ATP-binding capability of CMPK2.

To further verify the consequence of the above mutations, we overexpressed Flag-tagged variants of CMPK2 (ΔN26 (c.1 A > C and c.2 T > C), and Y414C (c.1241 A > G)) in cultured cells. Immunofluorescence analysis in Cos-7 cells using antibodies against Flag and the mitochondrial marker COX IV revealed that WT CMPK2 and CMPK2-Y414C, but not CMPK2-ΔN26, localized to the mitochondria (Fig. 1e). Western blotting of the mitochondrial fraction from primary cultured rat neurons transfected with Flag-tagged CMPK2-ΔN26 also confirmed the disruption of mitochondria localization (Fig. 1f, g). Interestingly, all the three Flag-tagged variants showed a marked reduction of CMPK2 protein expression levels compared to that of WT form, indicating that these mutations may also produce the nonsense-mediated mRNA decay (NMD) effect, which would exacerbate mutation-induced CMPK2 deficiency. These results support the notion that CMPK2 deficiency in its mitochondrial location and function may be the pathological basis of brain calcification in above families.

### Transcriptome analysis revealed abnormal energy metabolism of patients

**Fig. 2:**
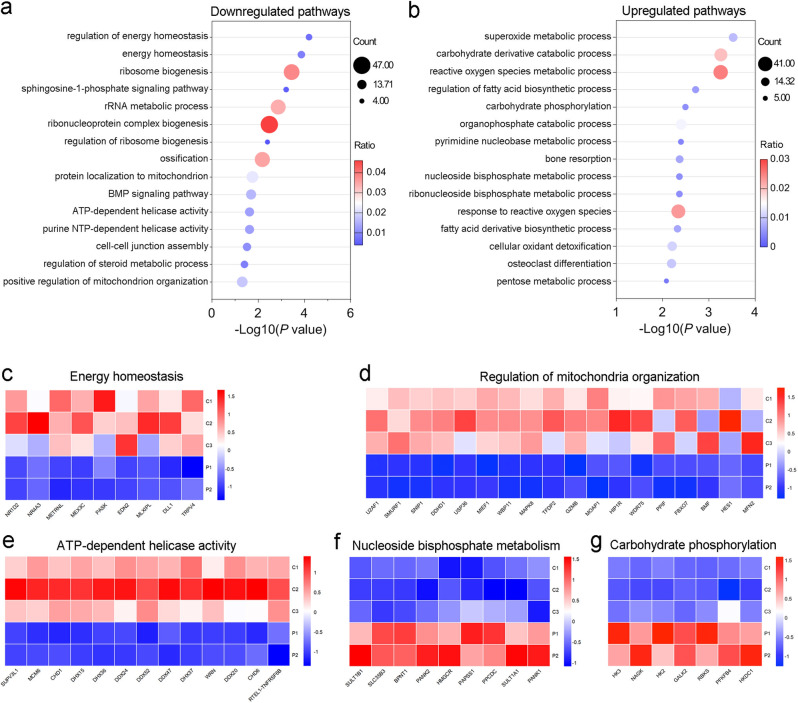
Transcriptome analysis of PBMCs. **a**, **b** RNA-sequencing analysis of PBMCs from the FBC patients compared with three unaffected controls (C1–C3). The upregulated (**a**) and downregulated (**b**) GO pathways were displayed. **c**–**e** Expression patterns of genes involved in representative downregulated pathways of PBMCs from patients and three unaffected controls, including energy homeostasis (**c**), regulation of mitochondria organization (**d**), and ATP-dependent helicase activity (**e**). **f, g** Expression patterns of genes involved in representative upregulated pathways of PBMCs from patients and three unaffected controls, including nucleoside bisphosphate metabolism (**f**) and carbohydrate phosphorylation (**g**).

To investigate whether the *CMPK2* mutations are bona fide to cause brain calcification in the families, we next carried out the transcriptome analysis of PBMCs to investigate the differentially expressed genes (DEGs) between patients (II-3 and II-5 in Family 1) and healthy controls. Compared to healthy controls, a total of 4579 uni-genes of patients were categorized as DEGs using DEG analysis (log2 fold change > 0, *P*-value < 0.05), with 2572 upregulated genes and 2007 downregulated genes. We next labeled the DEGs into sub-categories by GO term analysis. In the 241 downregulated biological process (BP) catalogues of GO, we noticed that the top two sub-categories were energy homeostasis and its regulation (Fig. 2a, c). Additionally, there are four reduced biological process related to ribosome biogenesis (e.g., ribosome biogenesis, rRNA metabolic process, ribonucleoprotein complex biogenesis, regulation of ribosome biogenesis). It is widely identified that homeostatic change of ribosome-related pathways is the major response to mitochondria function insufficiency^23,24^. Regulation of steroid metabolic process was also impaired in the patients, in accordance with the critical role of mitochondria in the synthesis of steroids^25,26^. Moreover, several other pathways related to mitochondrial function were also downregulated in the patient group (e.g., ATP-dependent helicase activity, purine NTP-dependent helicase activity, positive regulation of mitochondrion organization) (Fig. 2a, d, e). In response to these downregulation pathways, several energy metabolism-associated pathways, such as superoxide metabolic and ROS response, carbohydrate derivative catabolic process, fatty acid biosynthetic regulation, and nucleoside bisphosphate metabolism, were reciprocally up-regulated (Fig. 2b, f, g). Conclusively, the obvious energy metabolism dysregulation reflected by transcriptome analysis from patients’ PBMCs provides a strong indication for the association of mitochondrial dysfunction with brain calcification in our FBC patients, and further suggests that the *CMPK2* loss-of-function mutations may be the key driving genetic factor underlying the above-described symptoms.

### *Cmpk2* expression in mouse brain

**Fig. 3:**
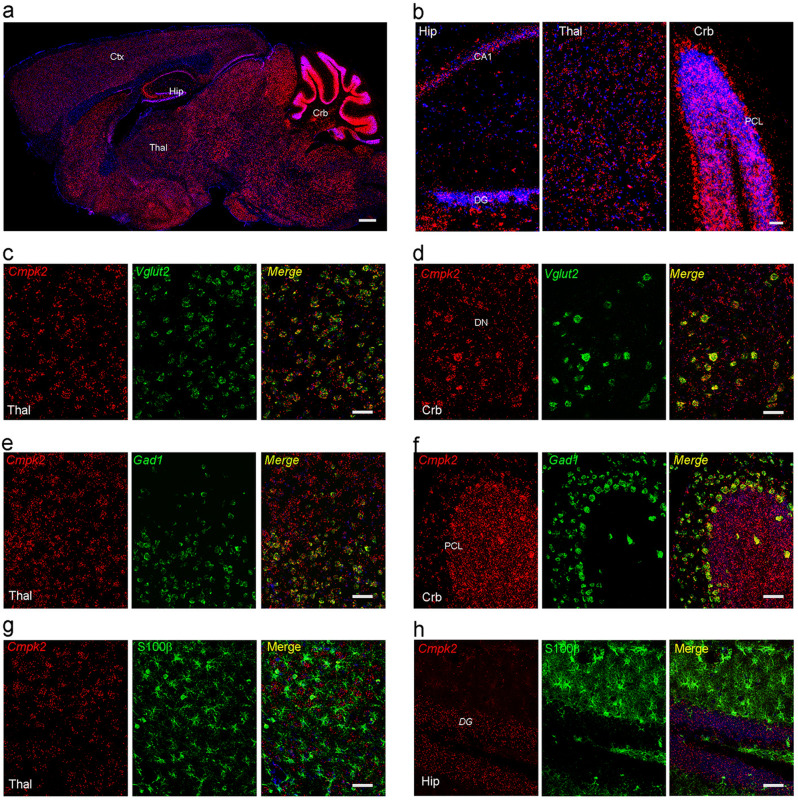
*Cmpk2* is expressed in mouse neurons. **a** In situ hybridization of *Cmpk2* expression in adult mouse brain sagittal sections. **b** Enlarged view of *Cmpk2* expression in the hippocampus, thalamus, and cerebellum; DAPI (blue) staining of nuclei. **c**, **d** Dual in situ hybridization of *Cmpk2* (red) with the glutamatergic neuronal marker *Vglut2* (green) in the mouse thalamus (**c**) and dentate nuclei of the cerebellum (**d**). **e**, **f** Dual in situ hybridization of *Cmpk2* (red) with the GABAergic neuronal marker *Gad1* (green) in the mouse thalamus (**e**) and the PCL of the cerebellum (**f**). **g**, **h** Double fluorescence labeling of *Cmpk2* (red) and the astrocytes marker S100β (green) in the thalamus (**g**) and DG of hippocampus (**h**). Ctx cortex, Hip hippocampus, Thal thalamus, Crb cerebellum, DG dentate gyrus, PCL Pukinje cell layer, GCL granule cell layer. Scale bars, 500 μm in **a**; 50 μm in **b**–**h**.

The human gene *CMPK2* is located on chromosome 2:6,840,570-6,866,635 (GRCh38) and encodes a protein with 449 amino acids designated as UMP-CMP kinase 2; it functions as a monophosphate kinase, and participates in the salvage pathway of dUTP and dCTP production, which are required for mitochondria genome DNA replication^21^. To further address the functional role of Cmpk2, the expression pattern of *Cmpk2* mRNA in WT mice brains was analyzed using in situ hybridization. Single probe hybridization showed that *Cmpk2* was expressed ubiquitously in all brain regions (Fig. 3a). Robust *Cmpk2* signals were enriched in the soma layer of Cornu Ammonis 1 (CA1) pyramidal neurons and in the granule cell layer (GCL) of the dentate gyrus (DG) of the hippocampus, as well as in the Pukinje cell layer (PCL) and the GCL of the cerebellum (Fig. 3b), suggesting enrichment in neurons.

To specify the identities of *Cmpk2*-expressing cells, we next employed dual hybridization combining the *Cmpk2* probe with other probes targeting cell-type marker genes. Notably, *Cmpk2* was co-labeled with glutamatergic neuron-specific *Slc17a6 (Vglut2)*-positive cells in the thalamus and in dentate nuclei of the cerebellum (Fig. 3c, d). Moreover, *Cmpk2* signals also co-localized with GABAergic neuron-specific *Gad1*-positive cells both in the thalamus and the PCL of the cerebellum (Fig. 3e, f). In contrast, no evident *Cmpk2* signal was detected in cells immune-stained positive for the astrocyte marker protein S100β (Fig. 3g, h). Further, based on published single-cell transcriptome data, we found that *Cmpk2* was also not apparently expressed in pericytes, vascular smooth muscle cells (vSMCs), or microglia (http://betsholtzlab.org/VascularSingleCells/database.html) (Supplementary Fig. S4a). Comparatively, robust expression of Cmpk2 in vascular endothelial cells (vECs) was found in this single-cell RNA-seq study, which was again confirmed by another separate study (http://celltypes.org) (Supplementary Fig. S4b). Collectively, the strong expression of *Cmpk2* in neurons and brain vECs supports that *Cmpk2* deficiency may cause brain disorder, including brain calcification.

### *Cmpk2* deficiency reduces mtDNA copy number and impairs the mitochondrial Pi homeostasis in mouse neurons

**Fig. 4:**
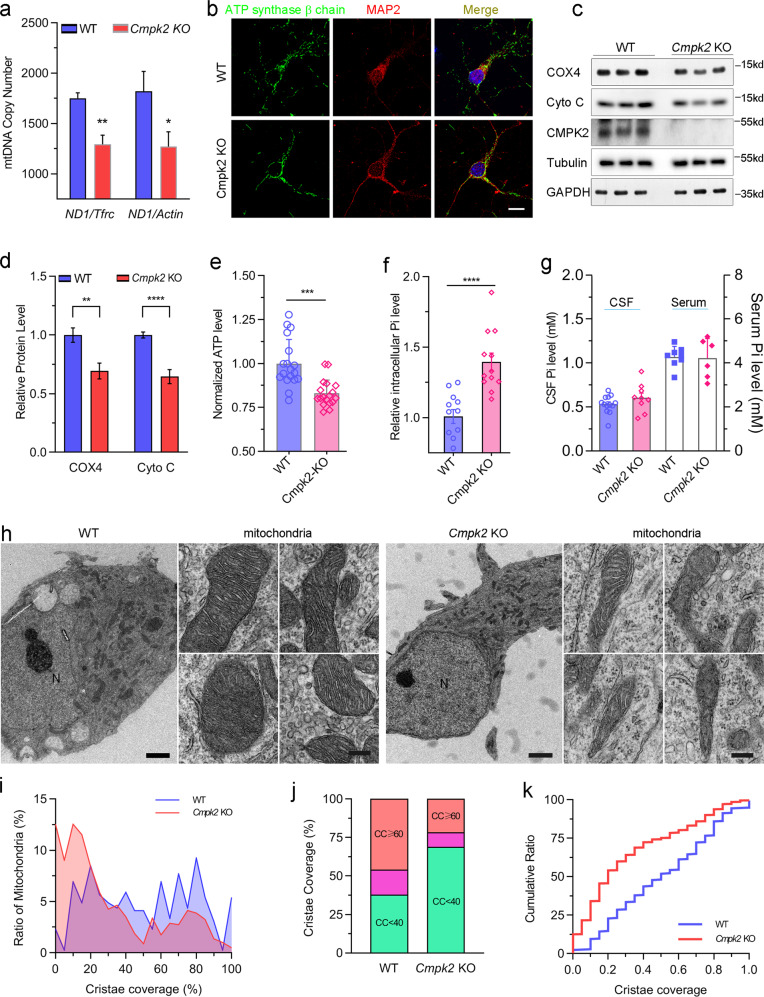
Cmpk2 deficiency impairs mitochondrial function in mouse neurons. **a** ddPCR-based analysis of mtDNA copy number between WT and *Cmpk2*-KO mouse cortex tissues. *n* = 7~8, three replicates. **b** Representative images of mitochondria in the primary cultured cortical neurons of WT and *Cmpk2*-KO mice with antibodies against the mitochondrial marker ATP synthase β chain (green) and the cytoskeleton protein MAP2 (red); DAPI (blue) was used for nuclear staining. Scale bar, 5 μm. **c** Western blot of COX IV and cytochrome C in the WT and *Cmpk2*-KO mouse primary cultured cortical neurons. **d** Quantification of the change in protein levels of COX IV and cytochrome C. *n* = 3, three replicates. **e** Measurement of ATP level of the WT and *Cmpk2*-KO mouse neurons. **f, g** The Pi level in the neuron (**f**), CSF and serum (**g**) in the WT and *Cmpk2*-KO mice. **h** Representative transmission electron micrograph of mitochondria morphology in the primary cultured cortical neurons of WT and *Cmpk2*-KO mice. **i**–**k** Statistical graphs of cristae coverage ratio of primary cultured cortex neurons in WT and *Cmpk2*-KO mice. CC cristae coverage. Error bars indicate means ± SEM. ns not significant. *P < 0.05, **P < 0.01, ***P < 0.001, ****P < 0.0001; Student’s *t*-test. Scale bars, 2 μm in **h**, each left panel and 0.2 μm in **h**, each right panel.

We generated *Cmpk2*-KO mice using one-step CRISPR-Cas9 strategy^21^. To evaluate whether Cmpk2 deficiency affects mtDNA synthesis, the mtDNA copy number of mouse brains was quantitatively analyzed using droplet digital PCR (ddPCR). Compared to WT littermates, the ratio of mtDNA (*Nd1*) to nuclear DNA (*β-actin* or *Tfrc*) in the brains of *Cmpk2*-KO mice exhibited a reduction of 25%–30% (Fig. 4a). As the cell type with high energy expenditure, neuron is more vulnerable to mitochondrial impairment. Considering that robust expression of *Cmpk2* was in neurons, and that *CMPK2* variants produced mitochondrial-targeting defect, we speculated that *Cmpk2*-deficient neurons may contribute to the brain calcification pathology. To verify this, we cultured neurons from *Cmpk2*-KO mice, and found that mtDNA-encoded respiratory chain enzyme complex IV (COX IV) and cytochrome C levels were evidently down-regulated (Fig. 4c, d). Immunofluorescence staining for the mitochondria marker ATP synthase β chain showed a reduction in its signal (Fig. 4b), morphologically supporting the decline of mitochondria pool in *Cmpk2*-KO mouse neurons.

The Cmpk2-related mitochondrial genome DNA synthesis dysfunction involves the abnormal phosphorylation for nucleotides in mitochondria. Indeed, we observed significant reduction of ATP level in the *Cmpk2*-KO mouse neurons (Fig. 4e), suggesting *Cmpk2*-KO does cause insufficient mitochondrial function. Moreover, we found that the intracellular Pi level of the *Cmpk2*-KO mouse neurons showed obvious elevation compared to that of WT littermates (Fig. 4f). However, no significant alteration of Pi concentration was found in the CSF and serum of *Cmpk2*-KO mouse (Fig. 4g). Therefore, these results demonstrate that loss of CMPK2 disrupts mitochondrial function and intracellular Pi homeostasis.

The mitochondria morphology of cultured primary neurons of WT and *Cmpk2-*KO mice was further evaluated by transmission electron microscopy (TEM). For context, cristae are specialized structures wherein respiratory chain super-complexes assemble to ensure high efficiency of energy production^23^; mitochondria in normal neurons have well-packed cristae that extend throughout the mitochondrial compartment (Fig. 4h). A large proportion of the mitochondria in neurons cultured from *Cmpk2-*KO mice had morphologically abnormal cristae (*e.g*., irregular packing, incompact and fragmented, warped cristae) and had obviously reduced compartment coverage; many of the mitochondria lacked any evident cristae (Fig. 4h). *Cmpk2*-KO neurons had a higher percentage of mitochondria with cristae coverage of less than 40% (Ratio: 68.8% in KO vs 37.8% in WT neurons, Fig. 4i–k), supporting that Cmpk2 somehow functions in maintaining normal neuronal mitochondria cristae morphology.

Taken together, these observations of mouse neurons demonstrate that a lack of Cmpk2 reduces mtDNA copy number and impairs phosphorus and energy homeostasis, suggesting that dysregulation of mitochondrial function may promote the initiation and progressive development of brain calcification.

### Calcification deposits in the brains of *Cmpk2-*KO and *Cmpk2-*KI mice

**Fig. 5:**
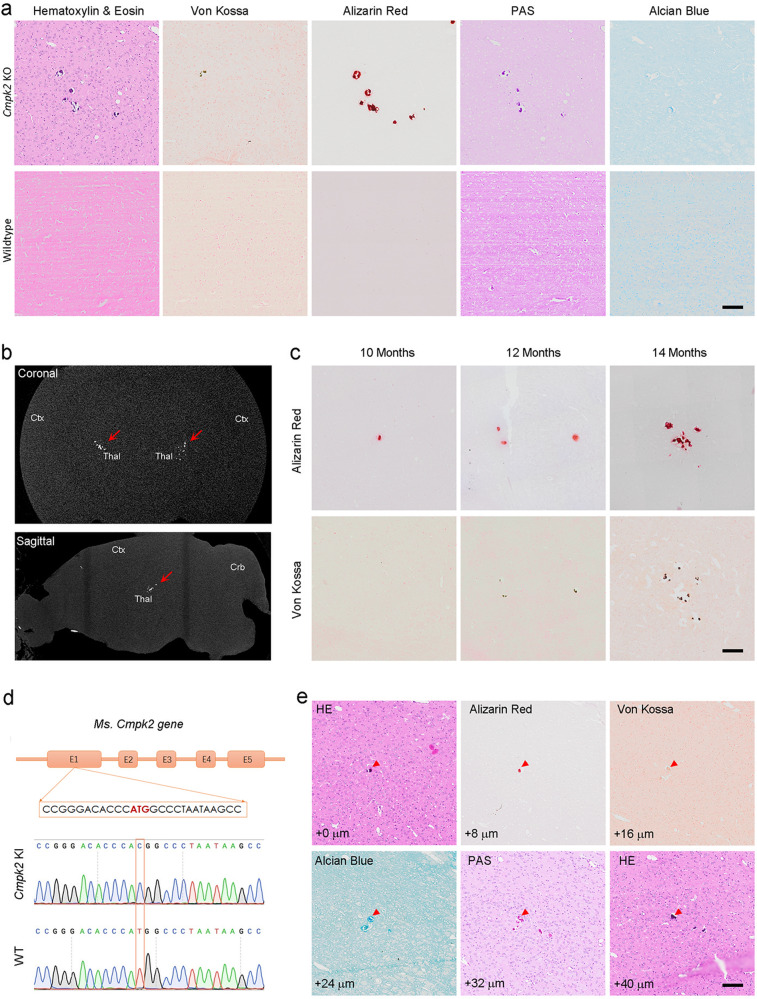
The brains of *Cmpk2*-KO and -KI mice have extensive calcification deposits. **a** Brain sections of 14-month-old WT and *Cmpk2*-KO mice were stained with H&E, Von Kossa, alizarin red, PAS, and Alcian blue. **b** Coronal and sagittal micro-CT scan of the brain of a *Cmpk2*-KO mouse at 13 months. Arrowheads indicate the high-density foci (these are calcification deposits). Ctx cortex, Crb cerebellum, Thal thalamus. **c** Progressive development of calcification deposits in the brains of *Cmpk2-*KO mice at 10, 12, and 14 months. Calcifications are indicated by alizarin red and Von Kossa staining. **d** A gene diagram for the *Cmpk2*-KI mouse model of the c.2 T > C mutation of the *Cmpk2* gene. **e** Brain sections of 12-month-old *Cmpk2*-KI mice were stained with H&E, Von Kossa, alizarin red, PAS, and Alcian blue. The calcification volume is indicated at the left bottom of a series of continuous brain sections. Scale bar, 100 μm in **a**, **c**, and **e**.

We periodically analyzed whether calcification deposits had developed in the *Cmpk2-*KO mice using micro-CT and multiple histological stains including alizarin red, Von Kossa, Alcian blue, and periodic acid-Schiff (PAS), as well as Hematoxylin and Eosin (H&E). At the age of 14 months, dense calcification deposits were observed in the thalamus of *Cmpk2-*KO (5 of 9 KO mice (age ≥ 10 months) showed calcification) but not WT mice (Fig. 5a). Moreover, temporal analysis of calcification progression showed that initial calcification deposits with single dots started at around 10 months and proceeded to multiple larger foci in the thalamus in mice examined at 12 and 14 months (Fig. 5c). Notably, the calcification nodules could be detected by micro-CT in 13-month-old *Cmpk2-*KO mice (Fig. 5b).

To further investigate the potential causal relationship of *Cmpk2* mutation and FBC, we generated a *Cmpk2*-KI mouse model by knocking in the patient mutation (c.2 T > C, p.M1?) into the corresponding locus of murine *Cmpk2* coding sequence. Similar to that in patients, this mutation leads to the loss of the mitochondrial-targeting peptide (mTP) and translation was predicted to be re-initiated by a subsequent ATG codon at p.M31 of murine Cmpk2 protein (Fig. 5d). Western blot analysis showed a moderately downward-shifted band of mutant Cmpk2 protein from homozygous *Cmpk2*-KI mouse brains. Without mTP motif, the mutant Cmpk2 protein was significantly diminished from mitochondria due to obvious cytoplasmic retention (Supplementary Fig. S6). Consequently, the homozygous *Cmpk2*-KI mice also developed obvious calcified deposits in the thalamus over 12 months (Fig. 5e), resembling that in *Cmpk2*-KO mice and *CMPK2-*variant carrying-patients. These results empirically confirm that *Cmpk2* deficiency is a causative factor for the formation of calcified deposits in the mouse brain, and further support that the *CMPK2* mutation can explain brain calcification phenotype in our FBC patients.

## Discussion

In this study we identified previously unreported *CMPK2* gene mutations that cause mitochondrial dysfunction to drive FBC pathogenesis. In both *Cmpk2*-KO and -KI mice, brain calcification was recapitulated in a progressive manner, mimicking the pathological development in human patients harboring a *CMPK2* mutations. Moreover, our observations of reduced mitochondria genome DNA copy numbers, down-regulated expression of mitochondrial proteins, reduced ATP production, abnormal intracellular Pi, and disrupted cristae in *Cmpk2*-deficient mouse brains, expands the pathological mechanism underlying brain calcification, which may facilitate the development of innovative therapies against brain calcification disorders.

Our findings broaden the scope of cellular mechanisms leading to brain calcification. Previously, three major pathogenic pathways were identified to drive FBC. First, impaired integrity of blood-brain-barrier (BBB) structure or reduced pericyte coverage results in increased BBB permeability; patients affected by this pathway may have mutations in genes such as in *PDGFRB, PDGFB, JAM2, or MYORG*^9–13^, which are all expressed by cells that constitute the neurovascular unit. The second pathway is the impairment of brain Pi homeostasis, either by hyperphosphatemia or by disrupted transport of phosphate in the brain; these patients suffering from this pathway defects may have mutations in *SLC20A2*, *XPR1*, or *GNAS* gene^7,8,27^ and usually show significantly increased CSF Pi levels. Immune dysregulation, especially in Aicardi-Goutières syndrome, triggers brain calcification by intracranial activation of the interferon pathway. Mutations in genes such as *ISG15* and *TREX1* have been linked to neuroinflammation-associated brain calcification^28^. In contrast, CMPK2 is enriched in the mitochondria of neurons or vECs, a distinct organelle and distinct cell types for brain calcification pathogenesis, and there is no obvious link for CMPK2 with the three aforementioned pathways. Thus, our results provide a novel line of support for the likely engagement of mitochondrial deficiency in the development of brain calcification. Previous analysis showed that brain calcification nodules were mainly surrounded by capillaries, astrocytes, and microglia. It is worth noting that our in situ hybridization results and publicly available scRNA-seq data revealed high-level expression of *Cmpk2* in both neurons and vECs, but at extremely low levels in vSMCs and in pericytes (*i.e*., the main type of mural cells) (Supplementary Fig. S4a). However, it bears emphasis that Cmpk2 expression could be upregulated in response to neuroinflammation stimulation^29^. Therefore, the contribution of other cell types, especially vECs, could not be neglected.

Although brain calcification was also reported as a rare pathological manifestation in mitochondriopathies^30^, they are different from the currently identified *CMPK2*-mutant cases. It is estimated that only 13.5% of patients with mitochondrial encephalomyopathy have bilateral brain calcification, suggesting low correlation of brain calcification with general mitochondria pathology. Brain calcification has been repeatedly reported in patients suffering from three syndromes: mitochondrial myopathy, encephalopathy, lactic acidosis, and stroke-like episodes (MELAS) syndrome^16^, Kearn-Sayre syndrome (KSS)^17^, and Leigh syndrome^18^. Many MELAS-associated genes are present in the mitochondrial genome (at least 17 genes)^31^, and therefore undergo only maternal inheritance; in contrast, the *CMPK2* locus is in the nuclear genome. Patients with MELAS syndrome show severe mitochondrial dysfunction that leads to multiple secondary symptoms^31^. Further, the blood or CSF lactate levels are significantly elevated in MELAS patients^32,33^, whereas our patient (II-5) had almost normal level of CSF lactic acid (2.2 mmol/L). Nor did our patients show any of the clinical manifestations linked to KSS or Leigh syndrome (e.g., progressive external ophthalmoplegia, extraocular muscles paralysis, or respiratory abnormality). Moreover, our brain calcification patients exhibited moderate mitochondrial dysfunction. Depletion of mtDNA (> 50% reduction of mtDNA copy number) and mtDNA deletions are used as molecular biomarkers for mitochondriopathies, for example, patients with mutations in the mtDNA polymerase *POLG* showed a dramatic decline (~70%) in mtDNA copy number^34^, which was reported to manifest with brain calcifications^35^. However, only a mild reduction (~25%) of mtDNA copy number was detected in our *Cmpk2*-KO mouse model. Collectively, our patients with CMPK2 deficiency did not meet the mitochondria disease criteria (MDC) score^36^, so more cases of brain calcification with *CMPK2* mutations will be required to comprehensively elucidate the underlying neuropathology and its potential relationship with mitochondriopathies.

Our findings of the mtDNA copy number reduction, decreased ATP production, intracellular Pi dysregulation, and mitochondrial cristae disturbance in *Cmpk2-*deficient neurons provide possible novel mechanisms leading to brain calcification. These findings from the *Cmpk2*-KO mice were compatible with the PBMC transcriptome analysis of the FBC patients that revealed energy metabolism dysfunction. Reduced mtDNA synthesis caused by *Cmpk2* deficiency may result in insufficient mtDNA-encoding protein synthesis, and consequently result in inadequate energy production^37,38^. As mitochondrial cristae are specialized for efficient energy production, the disruption of cristae provides a strong indication that mitochondrial energy production is likely impaired^39^. It is worth mentioning that mitochondria are organelles in which various phosphate species are used in a variety of metabolic pathways^38^. For example, phosphate is an essential substrate for the synthesis of CDP/dCDP and UDP/dUDP, so deficiency for the converting enzyme CMPK2 could trigger homeostatic up-regulation of the intra-mitochondrial phosphate level. Moreover, insufficient generation of ATP may also stimulate a program to up-regulate the intracellular phosphate level. Considering that neuronal firing and adaptive activation of vECs involve active calcium dynamics in their mitochondria, long-term engagement of these phosphate-related homeostatic responses may somehow lead to accumulation and eventually precipitation of calcium phosphate in these two cell types. Therefore, CMPK2 deficiency-associated impairment of mitochondrial function deepens our understanding of the pathogenic mechanisms underlying brain calcification.

It is also interesting to consider the possible association of CMPK2 with the interferon pathway in the context of brain calcification. In the human genome, the *CMPK2* locus is positioned between the *RSAD2* and *NRIR* loci^40^. RSAD2 and NRIR are interferon-associated factors, and CMPK2 expression was also found to be regulated upon the stimulation with type I interferon^40,41^, suggesting possible interplay between CMPK2 and interferon responses. As interferon stimulation has been previously shown to promote the progression of brain calcification^42^, investigation of any involvement of CMPK2 in the interferon pathway in human or mouse brains may help expand our understanding of how CMPK2 deficiency causes brain calcification.

A recent study reported that CMPK2-dependent mtDNA synthesis enables NLRP3 inflammasome activation in macrophages^29^, and NLRP3 was also found to promote neuroinflammation in Parkinson’s disease and Alzheimer’s disease^43–45^. Based on our evidence of recessive brain calcification families and both KO and KI mouse models, our study supports that disruption of CMPK2 function leads to brain calcification. This could be interpreted to indicate that long-term suppression of CMPK2-associated NLRP3 inflammasome signaling might also disturb the mitochondrial signal network for nucleotide synthesis thereby causing intracranial calcified deposits. Moreover, it is notable that pharmacological manipulation of NLRP3 inflammasomes has received attention as a potential therapeutic intervention against cancer and multiple inflammatory diseases^46–49^; whereas our study suggests a possible risk with this strategy: the inhibition of NLPR3 upstream modulators in mitochondria may lead to mitochondrial dysfunction that could drive brain calcification. Accordingly, investigations of these innovative anti-inflammation therapies should consider monitoring for mitochondrion dysfunction and brain calcification. Whether *CMPK2*-deficiency affects interferon-related pathway(s) in the brain represents a fascinating question for future investigations.

## Materials and methods

### Patient inclusion and genetic analysis

In the present study, brain calcification patients were assessed for their TCS as previously described^2^. Among the 117 families and 365 sporadic Chinese Han patients with brain calcification, a total of 45 families and 334 sporadic patients were tested negative for the six previously reported PFBC disease-causing genes: *SLC20A2*, *PDGFRB*, *PDGFB*, *XPR1*, *MYORG*, and *JAM2* by Sanger sequencing. We then identified potential pathogenic variants of *CMPK2* in two FBC families and subsequently expanded our examination to the remaining 43 families and 334 sporadic patients, along with 500 ethnically matched healthy control individuals. Informed written consent was obtained from all participants prior to inclusion. This study was approved by the Institutional Ethnics Committee of the First Affiliated Hospital of Fujian Medical University (MRCTA, ECFAH of FMU [2019]198) and the Second Affiliated Hospital of Zhejiang University (I2019001149).

Human genomic DNA was extracted from peripheral blood leukocytes using standard methods. For WES, whole exon libraries and whole genome libraries were prepared using Agilent SureSelect All Exon 50 MB (V6) capture kits. Sequencing was conducted using the Illumina HiSeq 3000 platform with an average 100× coverage depth for WES and 30× for WGS. The sequenced reads were aligned to human genome build 38 (GRCh38) using the Burrows-Wheeler Aligner (BWA) software. Variants were described according to the Genome Analysis Toolkit (GATK) and were annotated with ANNOVAR. Variants with a frequency of < 0.01 in the ESP, ExAC, 10000 Genomes, and gnomAD databases were further screened as possible disease-causing mutations based on their inheritance patterns. Finally, we analyzed the disease-causing variants based on an autosomal recessive inheritance model. For *CMPK2-*targeting analysis, Sanger sequencing was used to examine the coding exons of the *CMPK2* locus and a *CMPK2* cDNA molecule reverse transcribed from *CMPK2* transcripts, present in whole blood leukocytes (NM_207315). See Supplementary Table S2 for primers used in this study. The CMPK2 protein structure analysis was performed by PyMOL software.

### Transcriptome sequencing of human PBMCs

Total RNA was extracted from patients’ and healthy controls’ PBMCs using TRIzol (Invitrogen, USA) according to manufacturer’s instructions, followed by DNase I treatment to remove DNA contamination.

For the library preparation and sequencing of RNA, the remaining mRNAs were processed using the TruSeq RNA Sample Prep Kit according to the Illumina protocol. Then, mRNA library was constructed and quality tested according to the manufacturer’s instructions. The RNA library was sequenced on an Illumina Hiseq 4000 platform, and 150 bp paired-end reads were generated.

Data processing of raw reads was quality checked by using fastqc v0.10.1 (http://www.bioinformatics.babraham.ac.uk/projects/fastqc/) and trimmed for low-quality bases and adaptors if necessary by using TrimGalore (http://www.bioinformatics.babraham.ac.uk/projects/trim_galore/). Clean reads were mapped to the reference genome by using HISAT2 v2.0.4. The mapped reads of each sample were assembled by using StringTie v1.3.1 in a reference-based approach^50^.

Differential gene expression was analyzed using DESeq2 package and those genes with fold changes of the expression levels > 0 and *P*-value < 0.05 were identified as differentially expressed. The Database for Annotation, Visualization and Integrated Discovery (DAVID) and Gene Ontology resource were used to perform enrichment analysis on the Differential Gene Expression analysis.

### Animals

*Cmpk2-*KO mice and c.2 T > C (p.M1?) KI mice were generated via a CRISPR/Cas9 approach, and genotyping of the founders was performed as previously described^11^. Briefly, for KO mice, sgRNAs and Cas9 mRNA (100 ng/μL) synthesized by MEGAshortscript^TM^ and mMESSAGE mMACHINE^TM^ T7 ULTRA Transcription Kits (Invitrogen) were co-injected into C57BL/6 J zygotes, which were then implanted into surrogate female mice at the blastocyst stage. For KI mice, donor oligos were co-injected with sgRNA and Cas9 mRNA. Founder mouse tail genomic DNA was extracted, and gene-edited *Cmpk2* genomic sequences were PCR-amplified and cloned into the T-Vector (Promega, A137A). At least 20 clones of each F0 mouse were sequenced to evaluate *Cmpk2*-KO . Only *Cmpk2-*KO F0 mice and homozygous *Cmpk2* c.2 T > C (p.M1?) KI homozygous mice were used for subsequent experiments. The sgRNA sequences, donor oligo sequence, and genotyping primers are listed in Supplementary Table S2.

All the P0 Sprague-Dawley (SD) rats were purchased from Shanghai SLAC Laboratory Animal for the mitochondrial fraction analysis of three candidate variants. All mice were maintained at the animal facility of the Center for Excellence in Brain Science and Intelligence Technology of the Chinese Academy of Sciences, Shanghai. Mice were housed with a standard 12-h light/dark cycle, with free access to standard rodent chow and water. A mixture of male and female mice were employed in all studies; no sex bias was observed. All experimental procedures were approved by the Institutional Animal Care and Use Committee of the Institute of Neuroscience of the Chinese Academy of Sciences.

### In situ hybridization

All the instruments and reagents were rinsed or pretreated with diethylpyrocarbonate (DEPC) before in situ hybridization. Two-month-old mice were perfused by 4% paraformaldehyde (PFA) and consecutively dehydrated with 15% and 30% sucrose to prepare brain slices of 20 μm thickness, which were then mounted on Superfrost glass slides (Fisher). For dual in situ hybridization, an RNAscope Multiplex Fluorescent Reagent Kit v2 (Advanced Cell Diagnostics, 323100) was used in combination with a *Cmpk2* probe C1, with or without *Gad1* C2 or *Slc17a6* C2 (*Vglut2*) probes. For double labeling of in situ hybridization coupled with immunofluorescence, the brain slices were first subjected to in situ hybridization with the *Cmpk2* probe, followed by incubation with an anti-S100β antibodies (1:300, ab868, Abcam). Whole brain in situ hybridization images were captured using a VS120 microscope with 40× objective lens (Olympus). For co-localization imaging, in situ hybridization slices were imaged on a 40× objective lens Olympus FV3000 laser confocal microscope, using a 488/468 nm laser and a *z*-interval of 0.4 μm. Slices from at least three mice were repeated for each in situ hybridization analysis.

### Plasmid constructs

A Flag-tagged human *CMPK2* cDNA sequence was cloned into the pCMV-Entry vector (Origene). Based on this template, point mutations of *CMPK2* were introduced using a Mut Express® II Fast Mutagenesis Kit V2 (Vazyme), according to the manufacturer’s instructions. The primers for the plasmid constructs are listed in Supplementary Table S2.

### Cell culture and transfection

Cos-7 cells were cultured using DMEM supplemented with 10% (vol/vol) fetal bovine serum (FBS), with or without coverslips, in 12-well plates (Corning). CMPK2 plasmids were transfected using Lipofectamine 3000 (Invitrogen), following the manufacturer’s instructions. Primary culture of cortical neurons and the electroporation were described as has been previously described^51^. In brief, the dissociated rat neurons were transfected by an AMAXA Nucleofector (Lonza), 6 μg of plasmids of CMPK2-Flag, CMPK2-c.1 A > C-Flag, CMPK2-c.2 T > C-Flag, or CMPK2-c.1241 A > G-Flag, along with GFP were co-transfected. And 1 × 10^7^ cells were used in the electroporation for the subsequent biochemistry assay.

### Cell immunofluorescence analysis

For cell immunofluorescence analysis, the coverslips were first fixed with 4% PFA for 15 min, followed by penetration and blocking with 0.1% Triton X-100 in PBS solution with 5% BSA for an additional 15 min. Next, the coverslips were incubated with primary antibodies against Flag (1:2000, F7425, Sigma), COX IV (1:2000, ab33985, abcam), ATP synthase (1:500, MAB3494, Sigma), and MAP2 (1:1000, sc20172, Santa cruz) at 4 °C overnight. The next day, the coverslips were washed three times with PBS, and then incubated with corresponding secondary antibodies (Alexa Fluor, Invitrogen) for 2 h at room temperature. Finally, the coverslips were stained with Hoechst (Beyotime), washed another three times with PBS, and mounted onto glass slides. Fluorescence images were taken using a 60× objective lens Olympus FV3000 laser confocal microscope, using a 488/468 nm laser and a *z*-interval of 0.4 μm. These imaging analyses were performed in triplicate.

### Western blotting

The primary cultured rat neurons (3 days in vitro, DIV 3) after electroporation were harvested using Cell Mitochondria Isolation Kit (Beyotime) according to the manufacturer’s directions. And primary mouse neurons (DIV 5) of WT and *Cmpk2*-KO mice were collected in lysis buffer with protease inhibitors, and the lysates were centrifuged at 14,000× *g* for 15 min. The protein exacts were separated by 13% SDS-PAGE gel and blotted onto PVDF membrane. Membranes were blocked with 5% no-fat milk and then probed with antibodies against Flag (1:5000, F1804, Sigma-Aldrich), CMPK2 (1:1000, PA5-59570, ThermoFisher), COX IV (1:2000, #4850, Cell Signaling), cytochrome c (1:250, sc13156, Santa Cruz), or GFP (1:3000, B-2, Santa Cruz), and the corresponding secondary HRP-conjugated antibodies (1:2000, Cell Signaling). Bands were visualized by ECL (TIANGEN) and measured by Image J software.

### mtDNA copy number analysis

Quantification of mtDNA copy number was performed by ddPCR. Briefly, the total DNA from brain tissues of *Cmpk2*-KO and WT mice were extracted (TIANGEN; Gene Tech). The calibrated DNA samples then underwent ddPCR reactions with TaqMan primer/probe sets using the QX200 AutoDG Droplet Digital PCR System (Bio-Rad), according to manufacturer’s protocol. The probes and primers for ddPCR were purchased (ThermoFisher, 4458367) or are listed in the Supplementary Table S2. The mtDNA copy numbers were determined by normalizing the levels of total mtDNA to genomic DNA (*Nd1*-ms/*β-actin* or *Nd1*-ms/*Tfrc*-ms). Each sample was analyzed in triplicate.

### Histology and micro-CT scanning

Mice were anesthetized using chloral hydrate and perfused by 4% PFA in PBS as previously described^52^. The brains of homozygous *Cmpk2*-KO and WT mice were taken through a sequential series of ethanol dehydrations before paraffin embedding, then were sliced to a thickness of 4 μm. Histology staining for the assessment of brain calcification was performed as has been previously described^11^, using alizarin red, Von Kossa, Alcian blue, and PAS, as well as H&E. For micro-CT scanning, three-dimensional images of mice brains were obtained via X-ray micro-CT scanning (Quantum GX, PerkinElmer); the paraffin-embedded mouse brains were evaluated in high-resolution mode with an X-ray tube voltage of 90 kV, a current of 88 μA, and a voxel size of 72 μm. Each analysis was performed in triplicate.

### TEM

Briefly, primary cultured mouse pyramidal neurons derived from P0–P1 WT and *Cmpk2*-KO mice were imaged by TEM. Neurons were plated in a Thermanox plastic coverslip (ThermoFisher) and fixed in 2.5% glutaraldehyde at DIV 7, post-fixed with 1% OsO_4_ (TED PELLA, 18451), and stained with 4% uranium acetate. Then, they were dehydrated through increasing concentrations of ethanol followed by propylene oxide and subsequently embedded in resin. Ultrathin sections were cut to 70 nm on an Ultramicrotome (EM UC7, Leica, German) via ultradiamond knife (Diatome, Switzerland) and mounted on copper grids. The sections were then viewed and photographed by TEM (JEM-1230, JEOL, Japan) with a CCD camera (Gatan Orius SC, 200 w, 2048 × 2048). Mitochondrial cristae were observed and the coverage area was evaluated by well-trained observers in a double-blind study. Briefly, we measured the length and width of the cristae-covered region in the mitochondria and calculated its area, as well as the mitochondria area. The ratio of cristae-covered area to the whole mitochondria area refers to mitochondria cristae coverage. Then we categorized the different types of mitochondria according to their cristae coverage like < 40%, 40%–60%, and > 60%. The mitochondrial morphology quantification analysis of primary cultured cortex neurons (from TEM images) examined 40 WT neurons and 48 *Cmpk2*-KO neurons in three independent experiments.

### Intracellular phosphorus and ATP measurement

For the measurement of intracellular phosphorus, primary cultured WT and *Cmpk2*-KO neurons were washed with phosphorus-free DMEM (11971025, Gibco) three times. Then cells were lysed by the phosphorus-free lysis buffer (20 mM Tris-HCl, pH 7.4, 150 mM NaCl, 1% TritonX-100). The lysates were then used for the detection of inorganic phosphate (303-0030, Innova Biosciences).

For the measurement of intracellular ATP, primary cultured WT and *Cmpk2*-KO neurons were plated in the 96-well cell culture plates. In DIV 6, the ATP level is detected by the luminescent assay (ab113849, Abcam) according to the manufacturer’s instructions.

### Statistical analysis

Data are presented as means ± SEM. Statistical significance was assessed by Student’s *t*-tests. Differences were considered statistically significant at < 0.05 and are denoted as follows: *P < 0.05, **P < 0.01, ***P < 0.001, and ****P < 0.0001.

## Supplementary information

Supplementary materials

## Data Availability

The corresponding authors are willing to provide the data related to this manuscript upon reasonable request.
